# Takotsubo cardiomyopathy in cancer patients

**DOI:** 10.1186/s40959-019-0042-9

**Published:** 2019-07-01

**Authors:** Aakash Desai, Arish Noor, Saurabh Joshi, Agnes S. Kim

**Affiliations:** 1 Department of Medicine, University of Connecticut School of Medicine, Farmington, CT, USA; 2 Department of Medicine, Division of Cardiology, University of Connecticut School of Medicine, 263 Farmington Avenue, Farmington, CT 06030-2202 USA

**Keywords:** Chemotherapy, Takotsubo cardiomyopathy, Stress cardiomyopathy, Cancer

## Abstract

**Background:**

Cancer is a chronic condition that induces significant emotional and physical stress, which may increase the risk for developing Takotsubo cardiomyopathy (TCM).

**Main body:**

Takotsubo cardiomyopathy, also known as stress cardiomyopathy, is a clinical syndrome that generally presents as chest pain mimicking acute coronary syndrome or as an acute heart failure characterized by severe left ventricular systolic dysfunction in response to emotional, physical, or medical stress. The potential triggers for Takotsubo syndrome in cancer patients include the emotional turmoil of a cancer diagnosis, the inflammatory state of the cancer itself, and the physical stress of cancer surgery, systemic anti-neoplastic therapy, and radiation treatment. TCM is becoming increasingly recognized among patients with cancer and has been associated with adverse outcomes in this patient population. In this study, we searched the Pubmed database using keywords “Takotsubo cardiomyopathy”, “cancer”, and “anti-neoplastic therapy” to review case reports of Takotsubo syndrome occurring in oncologic patients after systemic anti-neoplastic therapy. Clinical presentation, electrocardiogram, laboratory data, transthoracic echocardiogram and coronary angiogram results, and patient outcomes were collected and analyzed.

**Conclusion:**

Patients with cancer are at an elevated risk for developing stress cardiomyopathy, and it is important to know which cancer drugs have been associated with the development of the Takotsubo syndrome.

## Background

Patients with cancer who are undergoing systemic therapy for their malignancy often have co-existent cardiovascular illness and/or risk factors. Physicians sometimes face difficulty in differentiating chemotherapy-induced cardiotoxicity from cardiac events unrelated to cancer treatment. Recognition of chemotherapy-induced cardiotoxicity is important since repeated administration of the offending drug can potentially lead to irreversible cardiac damage. On the other hand, premature discontinuation of an effective anti-neoplastic agent due to co-existing cardiac events not directly related to therapy may increase oncologic morbidity and mortality. One cardiac condition that may be directly caused by chemotherapy or may be completely unrelated to it is Takotsubo cardiomyopathy (TCM).

TCM is a clinical syndrome that generally presents as chest pain mimicking acute coronary syndrome (ACS) or as an acute heart failure characterized by severe left ventricular (LV) systolic dysfunction in response to emotional, physical, or medical stress. Unlike in ACS, patients generally have a normal coronary angiogram, the LV dysfunction extends beyond a coronary distribution and usually recovers within days or weeks. The most common pattern of LV dysfunction is apical akinesis or ballooning with hyperdynamic basal segments [1]. The pathophysiology of the syndrome is not well understood; however, catecholamine excess, coronary artery vasospasm, and microvascular dysfunction are thought to be the key mediating processes [2]. These mechanisms can be activated by various stressors, which include emotional or psychological stress, infection, surgery, medications, and exacerbation of chronic diseases [3]. Furthermore, a genetic predisposition for TCM has been postulated given the preponderance of familial cases [4].

More recently, anti-neoplastic agents have been associated with TCM. Certain cancer drugs have been implicated in triggering this form of cardiomyopathy. While studies regarding the cardiotoxic effects of chemotherapeutic agents are plentiful, the data on this type of cardiomyopathy induced by cancer treatment are sparse, and no known correlation between dose of chemotherapy and TCM currently exists. Our review explores the possible etiologic link between various cancer drugs and TCM. The cancer therapies that will be discussed include traditional chemotherapeutic agents as well as newer targeted drugs, including immune checkpoint inhibitors. With this review, we hope to shed light on the association between TCM and cancer as well as TCM and anti-neoplastic agents.

### Takotsubo cardiomyopathy

TCM was first described in Japan in 1990 by Dote and colleagues [1]. The particular name “Takotsubo” was used to describe the cardiomyopathy due to the characteristic apical ballooning on left ventriculography which was similar in shape to a Japanese octopus trap. It is also referred to as “broken heart”, “apical ballooning syndrome”, “myocardial stunning”, or “stress cardiomyopathy”. TCM may occur in the presence of an emotional or physiologic stressor.

The syndrome has been observed most frequently in post-menopausal women who are exposed to major stressors. Patients can experience symptoms varying from chest pain (63%) to dyspnea on exertion (8%) and syncope (3%) [3]. This poses an added diagnostic challenge since this constellation of symptoms can mimic myocardial infarction (MI), pulmonary embolism or cerebrovascular disorders. Patients can present with variable ECG changes that affect the ST segment and T wave [5]. ST segment elevation or new LBBB was present in 34.2% of cases, while T wave inversion and non-specific ST-T wave changes were seen in 30.4 and 35.2% respectively [5]. Mild elevation in cardiac enzymes may also be present; however, the degree of elevation is disproportionately low to the area of myocardium affected. Coronary angiography does not show obstructive CAD (defined as > 50% narrowing of the coronary artery) [2].

**Fig. 1:**
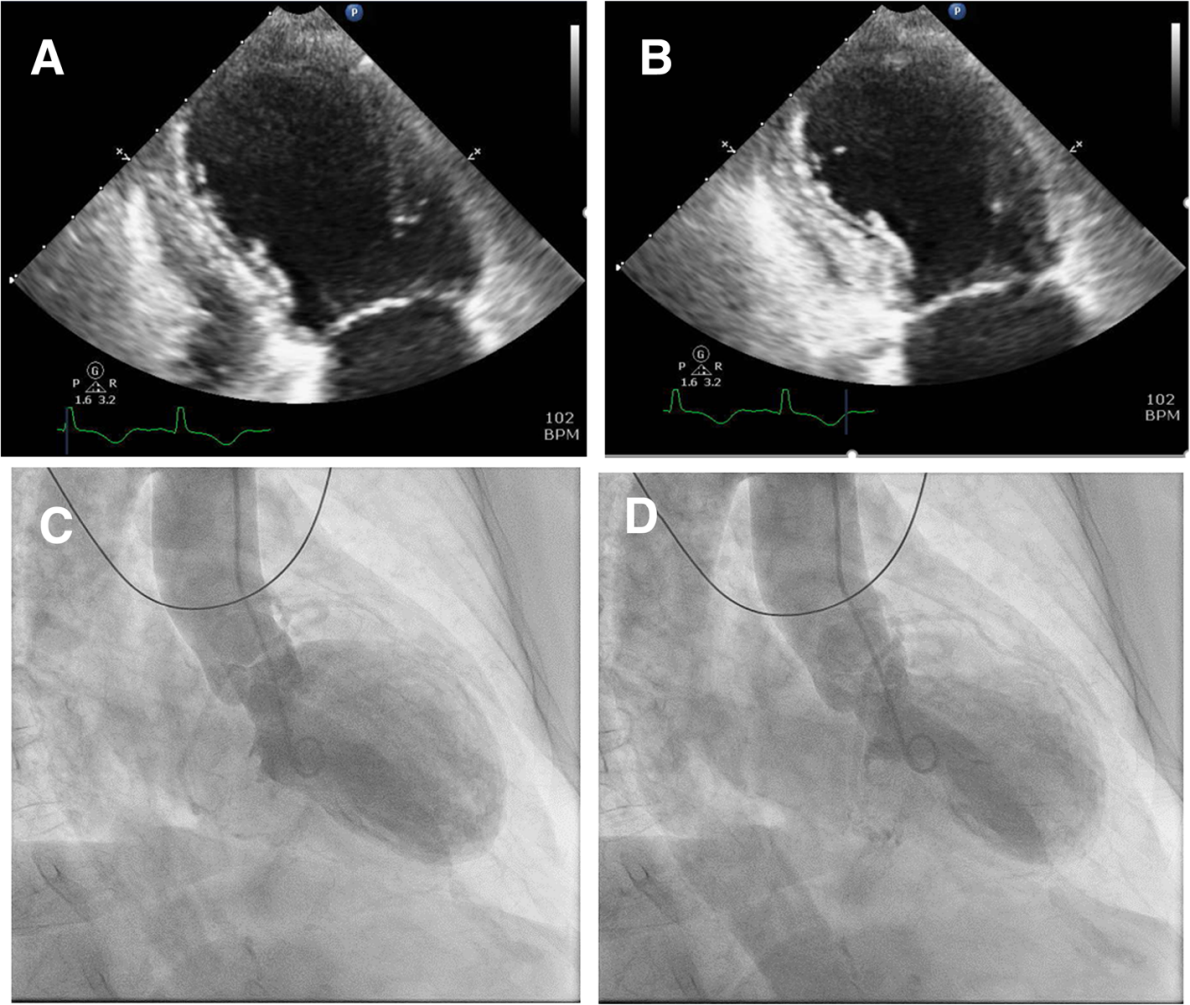
Echocardiogram pattern in a patient with TCM demonstrating apical ballooning and diffuse hypokinesis with sparing of the basal myocardial segments. Left ventricle in (**a**) end-diastole and (**b**) end-systole is shown; Left Ventriculogram pattern in TCM shown in (**c**) end-diastole and (**d**) end-systole

LV dysfunction is observed on imaging modalities like echocardiography and left ventriculography (Fig. 1). Three different types of contractile abnormality of the LV have been described. The most common pattern is apical akinesis with hyperkinesis of the basal segments. Other less common patterns include: combined akinesis of the mid-LV and apex, isolated akinesis of the mid-LV, and isolated basal akinesis [6]. Typically, LV ejection fraction (LVEF) is severely reduced, with median LVEF of 20%; interquartile range, 15 to 30% (normal LVEF range 55–75%) [2]. In a majority of patients, LVEF improves to greater than 50% on follow up. While cardiac recovery usually occurs in 4 to 6 weeks, it can take anywhere from 2.5 months to a year for a small proportion of individuals (~ 5%) [2, 7]. The prolonged duration required for normalization of LV systolic function predisposes individuals to the development thrombus in the LV with increased risk of embolization. Although uncommon, TCM can lead to complications like hemodynamic instability, atrial or ventricular arrhythmias, progressive heart failure, cardiogenic shock, and death. To aid in the diagnosis, “Mayo Clinic criteria” [8], can be used (Table 1)*.*

**Table 1:** Mayo Clinic diagnostic criteria for Takotsubo cardiomyopathy [8]

*For the diagnosis of TCM, the “Mayo Clinic criteria” must be met, which include* [8]:	
*1. Transient hypokinesis, akinesis or dyskinesis of left ventricular mid segments with/without apical involvement, regional wall motion abnormality extending beyond a single vascular territory, and a stressful trigger is often, but not always, present;* *2. Absence of angiographic evidence of obstructive coronary disease or plaque rupture;* *3. New ECG abnormalities consisting of ST elevation or T wave inversion with modest elevation in cardiac troponins;* *4. Absence of pheochromocytoma and myocarditis*	

Beta blockers along with ACE-inhibitors form the mainstay of treatment by reducing catecholamine stimulation and countering one of the main pathogenetic pathways. In-hospital mortality can be as high as 16% [9]. Given the paucity of lengthy follow-up studies, data regarding long-term prognosis and outcomes are currently lacking.

### Pathogenetic mechanisms of Takotsubo cardiomyopathy

**Fig. 2:**
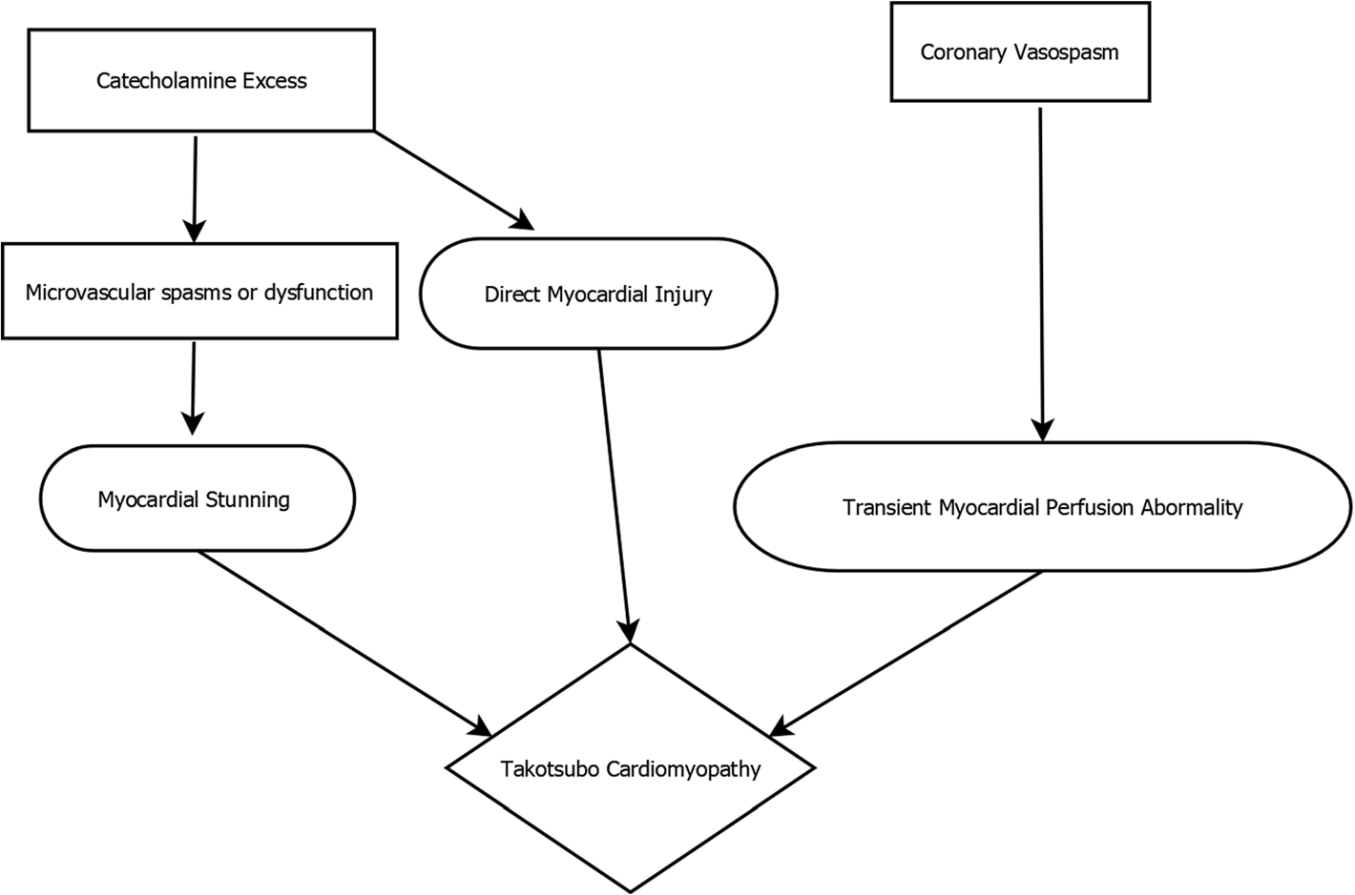
Proposed Pathogenetic Mechanisms of TCM

The exact pathophysiology behind TCM is currently unknown. The rationale behind increased incidence in postmenopausal women or the predilection for the LV apex or mid-cavity are currently unanswered questions. Various postulated mechanisms of TCM include: catecholamine excess, coronary artery vasospasm, microvascular dysfunction and upregulation of certain cardiac genes (Fig. 2).

Catecholamines released during a stressful event play a significant role in the development of cardiomyopathy. Wittstein et al. [2] found that patients with LV dysfunction after emotional stress had elevated catecholamines. The pivotal role of catecholamines is also supported by Abraham et al. [10] where TCM was induced after infusion of norepinephrine and dopamine. The stimulation of cAMP increases the intracellular concentration of norepinephrine within the cardiac myocytes, which can lead to damage. The key role of norepinephrine is also supported by the fact that the use of beta-blockers can significantly reduce damage. The findings of multifocal coronary vasospasm and transient myocardial perfusion abnormalities also suggest coronary artery vasospasm as one of the mechanisms inducing TCM. Drugs have been implicated as a cause of TCM, particularly in those situations in which no clear emotional or other stress trigger could be identified [11].

## Takotsubo cardiomyopathy and cancer

**Fig. 3:**
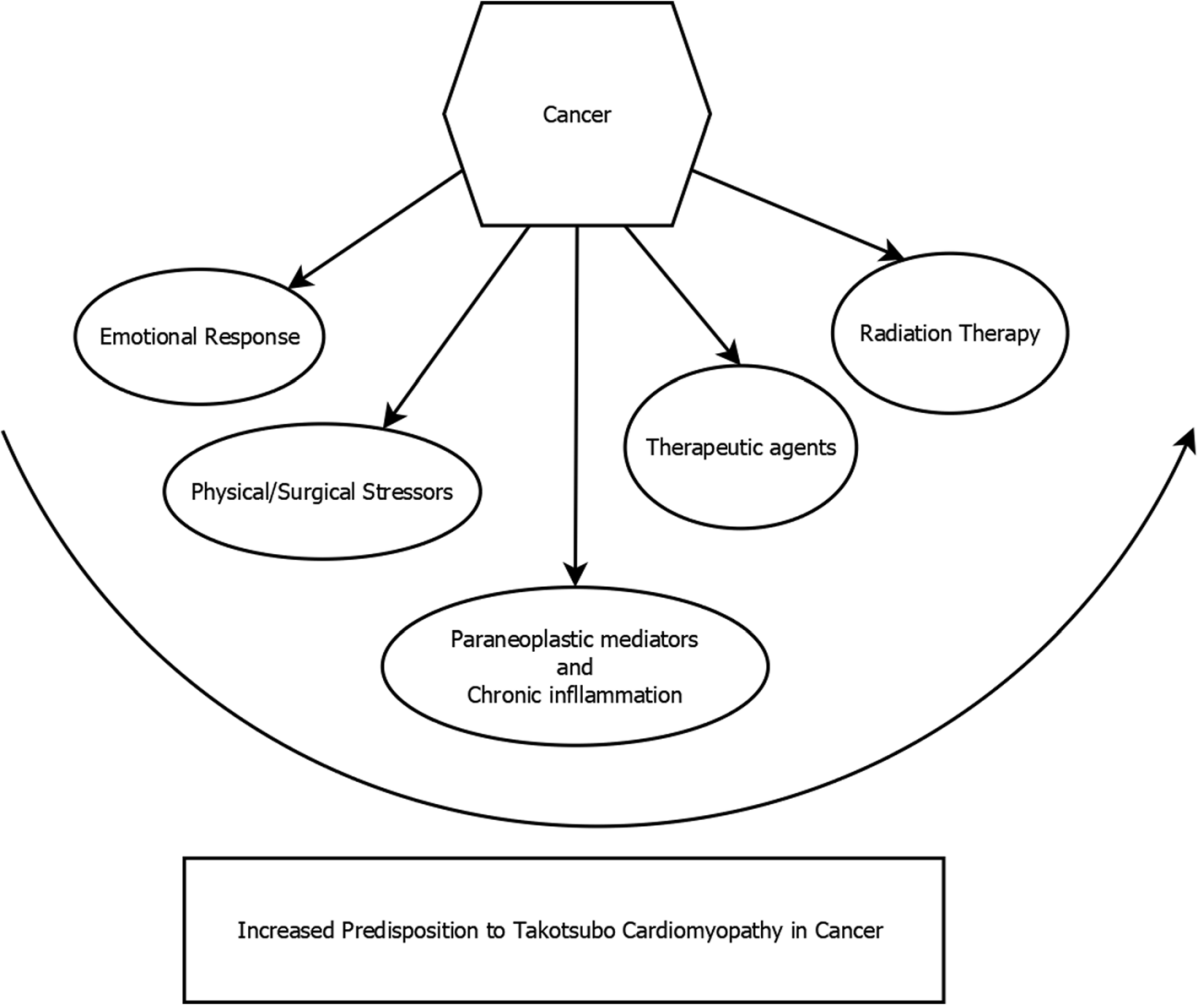
Cancer and Takotsubo cardiomyopathy: Cancer can increase the predisposition to TCM through various pathways. Cancer produces an emotional stress response as well as physical/surgical stressors from the disease. The paraneoplastic mediators along with the chronic inflammatory state is another risk factor. Lastly, the therapeutic regimens including medical therapeutic agents and radiation therapy can trigger the development of TCM

Cancer is a chronic condition that induces significant emotional and physical stress, increasing the risk of stress cardiomyopathy. The potential triggers for TCM in cancer patients include emotional turmoil of the diagnosis, the inflammatory state of cancer, and the physical stress of various cancer treatments, including chemotherapy (Fig. 3) [12, 13]. In addition, it has been hypothesized that the circulating paraneoplastic mediators may modify the adrenoreceptors in cardiac tissue, leading to contractile dysfunction.

The cardiotoxicity of chemotherapeutic agents, such as anthracyclines and trastuzumab, is a well-known entity among cardiologists and oncologists alike [14]. The wide range of potential cardiac side effects rising from cancer therapy include ventricular dysfunction, ischemia, arrhythmia, hypertension, accelerated atherosclerosis, venous thromboembolism, myocarditis and QT prolongation [15–17]. Several anti-neoplastic agents have been implicated to be associated with TCM. Among these drugs, the most commonly reported is 5-Fluorouracil. Other cancer therapies that have been reported to induce this type of cardiomyopathy include capecitabine, combretastatin, rituximab, vascular endothelial growth factors inhibitors, other angiogenesis inhibitors, taxols and others (Tables 2 and 3). TCM is probably an under-recognized and thus under-diagnosed disease entity among cancer patients receiving chemotherapy [12].

**Table 2:** Case reports of cancer drugs reported to cause Takotsubo Cardiomyopathy with details of the case (cancer drug, type of cancer, patient demographics, presence or absence of chest pain, and results of cardiac testing)

Reference No.	Age of patient	Sex	Known CAD?	Cancer	Chemotherapy	Time of TCM Onset	Chest Pain	ECG abnormality	Peak Troponin levels in ng/ml (Normal Value Reported)
[18]	60	F	No	Stage III colorectal cancer	Continuous 5- FU infusion [as part of FOLFOX]	First cycle	Yes	Sinus Tachycardia; STE in II,III,aVF, V4,V5,V6	Trop-I = 0.38 (N/A)
[19]	62	F	No	Rectal adenocarcinoma	5 FU + Levofolinate	Sixth cycle	Yes	STE in V1-V3	Trop-T = 0.82 (N/A)
[20]	58	F	No	Metastatic adenocarcinoma of the colon	FOLFOX (5 FU, Leucovorin, Oxaliplatin)	First cycle	No	Non specific lateral ST changes and poor R wave progression	Trop-T = 0.64 (< 0.03)
[21]	52	M	No	Squamous cell carcinoma of soft palate	Cisplatin + 5-FU	First cycle	No	Sinus tachycardia; LVH with repolarization changes	N/A
[17]	54	M	N/A	Metastatic adenocarcinoma of the colon	FOLFOX (5 FU, Leucovorin, Oxaliplatin)	First cycle	Yes	Lateral STE with reciprocal changes with intermittent LBBB	Trop-I = 4.07 (N/A)
[22]	48	M	No	Adenocarcinoma of the colon	FOLFOX (5 FU, Leucovorin, Oxaliplatin)	First cycle	Yes	Apicolateral STD and TWI	Trop = 0.5 (< 0.1)
[23]	59	M	N/A	Invasive adenocarcinoma of sigmoid colon	FOLFOX (5 FU, Leucovorin, Oxaliplatin)	First cycle	Yes	Upsloping STE and hyperacute T wave changes in septal, inferior, and lateral leads	Trop-I = 1.0 (< 0.04)
[24]	48	M	No	Gastric adenocarcinoma	DCF (Docetaxel, Cisplatin, 5-FU) followed by FOLFIRI (5-FU, Folinic acid, Irinotecan)	Seventh cycle	No	Sinus tachycardia; TWI in V4,V5	Trop-I = 2.87 (< 0.04)
[25]	14	M	No	Metastatic nasopharyngeal carcinoma	Cisplatin + 5-FU infusion	First cycle	No	Sinus tachycardia with no ST-T changes	N/A
[26]	79	F	No	Metastatic adenocarcinoma of the Colon	5 FU	Tenth cycle	Yes	STE in anterior and inferior leads	Trop-T = 1.06 (< 0.04)
[20]	81	F	No	Stage III colorectal cancer	5-FU + Folinic Acid switched to Capecitabine	5 cycles of 5-FU, First cycle of Capecitabine	Yes	TWI in anteroseptal, inferior, and lateral leads	Trop-T = 0.35 (N/A)
[27]	47	F	N/A	Metastatic invasive ductal breast carcinoma	Capecitabine	First cycle	Yes	Nonspecific ST-T wave changes, followed by new STE in the inferolateral leads; STD and TWI in leads V1 and V2	Trop-I = 0.19 (0–0.75)
[28]	55	M	No	Adenocarcinoma of the caecum	Capecitabine	First cycle	Yes	1–2 mm STE in V2–6; 1 mm STD in aVR, II and aVF	HS Trop = 89 (N/A)
[29]	62	M	No	Adenocarcinoma of the colon	Capecitabine	First cycle	No	STE in precordial leads	N/A
[30]	39	F	No	Inflammatory breast cancer	Capecitabine	First cycle	No	Sinus tachycardia; TWI in anterolateral leads	Trop-I = 0.34 (N/A)
[31]	76	M	N/A	Colon cancer	Bevacizumab	First cycle	No	Anterior and inferior STE	N/A
[31]	61	M	N/A	Non small cell lung cancer	Bevacizumab	Second cycle	No	Tachycardia; new inferior Q waves; diffuse STE	Trop = 2.5 (N/A)
[32]	71	F	No	Anaplastic thyroid cancer	Combretastatin + Cisplatin + Doxorubicin	First cycle	No	TWI in I, aVL, V2- V6	Trop-I = 0.85 (< 0.15)
	78	F	No	Anaplastic thyroid cancer	Combretastatin + Cisplatin + Doxorubicin	First cycle	No	Deep symetric TWI in precordial leads	N/A
[33]	66	M	No	CLL	Rituximab + Methylprednisone	Thirteenth cycle	No	Sinus tachycardia; STE in I, II, V4–6	Trop = 0.14 (< 0.04)
[34]	57	F	No	Clear cell renal carcinoma	Sunitinib	N/A	Yes	STE	Trop-T = 1.2 (< 0.04)
[35]	71	F	No	Metastatic renal cell carcinoma	Axitinib	First cycle	Yes	Anterolateral STE	Trop-I = 6.95 (N/A)
[36]	50	F	No	Invasive ductal carcinoma of breast	Trastuzumab + Docetaxel + Carboplatin	Eleventh cycle	Yes	TWI in V1–2, I, aVL	Trop-T = 0.15 (< 0.02)
[37]	55	M	No	Non M3-AML	Cytarabine + Daunorubicin	First cycle (Day 6)	Yes	Sinus tachycardia; STE in I, aVL, V5, V6	Trop-I = 38.64 (< 0.1)
[38]	83	F	No	Metastatic melanoma	Ipilimumab	Fourth cycle	Yes	Sinus tachycardia; 1 mm STE in I, V2, V3	Trop-I = 0.98 (< 0.04)

**Table 3:** Reported nadir LVEF, wall motion abnormalities, follow-up LVEF, and patient outcome

Reference No.	Nadir LVEF (%)	LV Wall Motion Abnormality	Coronary Angiogram	Followup LVEF (At Interval)	Outcome
[18]	15–20%	Severe global hypokinesis with apical ballooning in systole and diastole	non-obstructive CAD	55–60% (4 weeks)	Survived
[19]	28%	Extensive apical akinesis	normal coronary arteries	67% (10 days)	Survived
[20]	15%	Severe diffuse hypokinesis	N/A	40–45% (10 days), 60% (3 months)	Survived
[21]	20%	N/A	N/A	60% (N/A)	Survived
[17]	30%	No regional wall motion abnormality	normal coronary arteries	55–65% (2 months)	Survived
[22]	15%	Severe hypokinesia in all apical and mid segments, normokinetic basal wall	N/A	30% (1 day), 55–65% (5 days)	Survived
[23]	10% (Cardiac Arrest)	Global Hypokinesia	non-obstructive CAD	68% (4 weeks)	Survived
[24]	15%	Hypokinesis of mid-apical and hyperkinesis of basal segments	non-obstructive CAD	50% (4 weeks)	Deceased (Progression of Cancer)
[25]	20%	N/A	N/A	EF recovered (2 weeks)	Deceased (Cardiac Asystole during 5th cycle of 5FU)
[26]	34%	Apical and periapical akinesia	non-obstructive CAD	70% (4 weeks)	Survived
[20]	35%	Akinesis of the distal half of the anterior and inferior walls not consistent with a single vascular territory	non-obstructive CAD	60% (4 weeks)	Survived
[27]	30%	Anteroapical wall motion abnormality	non-obstructive CAD	55% (6 weeks)	Survived
[28]	15–20%	Hypokinetic basal segment	Atheromatous changes with markedly sluggish blood flow in LAD	55% (1 week)	Survived
[29]	35%	Extensive anterior wall hypokinesis inconsistent with a single coronary artery territory	non-obstructive CAD	55–65% (1 week)	Survived
[30]	28%	N/A	N/A	62% (1 week)	Survived
[31]	N/A	Apical akinesis and a hyperdynamic basal segment	N/A	Normal (3 weeks)	Survived
[31]	N/A	N/A	non-obstructive CAD	Recovered (2 weeks)	Survived
[32]	40–50%	Apical akinesis	non-obstructive CAD	55–65% (4 weeks)	Survived
	50–55%	Apical/Septal hypokinesis	N/A	60–65% (4 weeks)	Survived
[33]	40%	Hypokinesis of the anterior wall	non-obstructive CAD	N/A	Death due to pathological fracture (unrelated to cardiomyopathy)
[34]	15–20%	Global hypokinesis with apical ballooning in systole and diastole	non-obstructive CAD	68% (3 months)	Survived
[35]	20–25%	Severe global hypokinesis with anterior apical ballooning	non-obstructive CAD	50–55% (3 weeks)	Survived
[36]	N/A	Systolic bulge at mid-LV with normal apical and basal segments	normal coronary arteries	Normal (6 weeks)	Survived
[37]	30–35%	Segmental wall motion abnormalities: mild anterior, septal, apical, inferior and lateral wall hypokinesia	non-obstructive CAD	50% (2 weeks)	Survived
[38]	50%	Akinetic apex, hyperkinetic base and septum	non-obstructive CAD	N/A	Survived

In this study, we searched the Pubmed database using keywords “Takotsubo cardiomyopathy”, “cancer”, and “anti-neoplastic therapy” to review case reports of Takotsubo syndrome occurring in oncologic patients after systemic anti-neoplastic therapy. Clinical presentation, electrocardiogram, laboratory data, transthoracic echocardiogram and coronary angiogram results, and patient outcome were collected and analyzed. What follows is a review of the anti-neoplastic drugs that have been reported to cause TCM.

## Anti-neoplastic drugs and Takotsubo cardiomyopathy

### 5-fluorouracil (5-FU)

5-Fluorouracil is a pyrimidine analog that inhibits the thymidylate synthase enzyme in malignant cells, causing disruption of DNA synthesis and resulting in cell death [22]. It is administered by intravenous injection as bolus or infusion in the cytostatic treatment of solid tumors, including colorectal, gastric, esophageal, pancreatic, prostate, bladder, and breast cancers. It is the foundation of adjuvant chemotherapy for colorectal cancer and is used in combination with irinotecan or oxaliplatin along with leucovorin as FOLFIRI (5-FU, folinic acid and Irinotecan) [39] or FOLFOX (5-FU, folinic acid and oxaliplatin) [40] regimens.

The most common cardiovascular side effect of 5-FU is chest pain with or without ECG changes. The incidence of cardiotoxicity ranges from 1.5–18% [41]. Clinical manifestations include signs of acute MI with anginal chest pain and ECG abnormalities, rhythm disturbances, heart failure, and a clinical picture consistent with TCM [21]. Basselin et al. [18] reported a likely causal relationship between 5-FU and TCM based on a score of 8 on the Naranjo adverse drug reaction probability scale (a probabilistic calculation of the likelihood of correlation between the adverse reaction and the drug administered) [19]. TCM caused by 5-FU may persist for up to 1 month following discontinuation of therapy [24, 25].

To date, 10 case vignettes have been published reporting TCM related to 5-FU administration [23, 26, 42–44]. The patients represented in the case reports range from 14 to 79 years of age [45, 46]. Although TCM generally occurs more frequently in postmenopausal women, TCM related to 5-FU had an incidence that was similar in both sexes. The majority of patients had colorectal cancer, and two cases occurred in the setting of head and neck cancer. In all the reported cases of TCM, 5-FU was used as part of primary chemotherapy regimen, e.g. FOLFOX, FOLFIRI or cisplatin-based regimen. In the majority of these cases, TCM occurred during or immediately after continuous infusion of 5-FU, with 7 out of 10 cases occurring during the first cycle. Most of these patients presented with chest pain. One presented with ventricular fibrillation cardiac arrest (who subsequently survived), and one experienced sudden circulatory collapse and death during the fifth cycle of 5-FU infusion following recovery from TCM during the first cycle [42, 43]. These 10 patients had varying degrees of ECG changes and had reduced LVEF ranging from 10 to 34%. None had obstructive CAD on coronary angiogram [47, 48]. All patients except the one who died had recovery of cardiac function with normalization of LVEF in 1–4 weeks.

Reintroduction of 5-FU following cardiotoxicity has been a topic of much debate. Successful re-challenge with 5-FU has been accomplished; however, a mortality rate of 13% has been seen with re-challenge [49]. Another study demonstrated that 18 out of 20 patients who underwent retreatment with 5-FU following cardiac side effects experienced further complications, including three MI and two deaths. Thus, the balance of evidence appears to argue against the reintroduction of 5-FU after recovery of cardiac function; however, a comprehensive risk-benefit assessment, extensive discussion with the patient, and multidisciplinary team approach is of paramount importance [50].

### Capecitabine

Capecitabine is an oral chemotherapeutic drug, which is activated preferentially in tumor tissue by enzymatic conversion to 5-FU [51]. With its introduction in 1990, it was hoped that given the preferential activation to 5-FU in cancer cells, capecitabine would have a favorable balance of efficacy and toxicity along with the ease of oral administration [52]. Since its inception, it has been used to treat a wide variety of tumors including breast, colorectal, and other gastrointestinal cancers [53].

Despite earlier beliefs, it is now understood that the cardiac side-effect profile of capecitabine is like that of 5-FU. A retrospective study including 1189 patients receiving either capecitabine or 5-FU reported an overall 3% incidence of cardiotoxicity. The incidence rates of cardiac side effects were similar in the capecitabine and 5-FU groups [20]. Cardiovascular side effects related to capecitabine include angina-like chest pain, MI, cardiogenic shock, and sudden cardiac death [54]. Of note, all patients had symptoms that started within 72 h of drug initiation and recovered within 6 weeks of drug cessation.

Coronary artery vasospasm, like that postulated for 5-FU, is the most commonly accepted hypothesis for capecitabine-induced cardiotoxicity. However, other explanations such as direct myocardial injury, thrombogenesis, and autoimmune etiologies have also been proposed [55].

To date, five cases of capecitabine-induced TCM have been reported in the literature. These cases occurred in patients between 47 and 81 years of age with a similar incidence between the two genders. These patients did not have a past history of CAD and were undergoing treatment of either colorectal cancer (3 cases) [20, 28, 29] or metastatic breast cancer (2 cases) [27, 30]. All of the TCM cases occurred during the first cycle of capecitabine treatment with the exception of one case that had received 5 prior cycles of 5-FU before being switched to capecitabine. All patients had ECG changes with elevated cardiac enzymes, normal coronary angiography, and a complete recovery of LVEF in 1–4 weeks.

### Bevacizumab

Bevacizumab is a monoclonal antibody whose antitumor activity depends on the inhibition of vascular endothelial growth factor (VEGF) signaling [56]. The role of VEGF is well-described in tumor growth and metastasis. In the healthy adult cardiovascular system, it is believed to contribute to vascular homeostasis and function [57]. Bevacizumab acts as an anti-neoplastic drug via multiple mechanisms including prevention of angiogenesis, facilitation of chemotherapeutic delivery, and reduced interstitial pressure of tumor. It is a novel cancer drug currently approved as part of combination therapy for metastatic colorectal cancer, non-small cell lung cancer, and breast cancer [58].

Bevacizumab has been associated with certain cardiovascular side effects, particularly arterial thromboembolism [59]. Other side effects include hypertension, cerebral infarction, transient ischemic attack, MI, and angina [60]. Studies on a genetically engineered mouse model that is capable of blocking the VEGF signaling pathway had increased dilation of the ventricles and reduced contractile function, leading to heart failure [31]. In addition, high concentrations of inflammatory cytokines and chemoattractant particles in myocardial tissue and plasma have also been proposed to cause cardiac damage [61].

To date, two cases of bevacizumab-induced TCM have been reported [60]. The first case reported a 76-year-old man with colon cancer, who developed anterior and inferior ST elevation 2 days after receiving bevacizumab. He had no significant CAD on angiography and had reduced LVEF with typical wall motion abnormalities of TCM. The other case vignette reported a 61-year-old man with non-small cell lung cancer who presented with hemoptysis and was found to have new Q waves in the inferior and precordial leads along with ST elevation. Coronary angiography did not show any significant disease, and the pattern of LV dysfunction was again classic for TCM. Both of these patients survived with complete recovery of LV systolic function within 2–3 weeks.

### Combretastatin

Combretastatin consists of the active compound combretastatin A4 phosphate (CA4P) derived from the African bush willow, Combretum caffrum, which possesses tumor vascular-targeting activity [62]. Its mechanism is based on depolymerizing the tubulin and is similar in structure to colchicine. It induces cytoskeletal changes in endothelial cells, increases vascular permeability, inhibits blood flow [63] and causes endothelial cell apoptosis [64]. It has demonstrated therapeutic benefit in anaplastic thyroid cancers [65] and medullary thyroid carcinomas [66]. CA4P-induced capillary and myocardial injury have been shown to occur due to collapse of the microcirculation in the myocardium. Moreover, CA4P has a direct toxic effect on cardiomyocytes [67].

Combretastatin-related TCM has been reported in two postmenopausal women between 71 and 78 years of age without any known history of CAD [68]. Both patients had anaplastic thyroid carcinoma where combretastatin was used as part of primary chemotherapy regimen with cisplatin and doxorubicin. The development of TCM was temporally associated with combretastatin administration and occurred during the first cycle. They presented without any complaints of chest pain, had varying degrees of ECG changes, and reduced LVEF ranging from 40 to 50%. Coronary angiogram, performed in only one patient, was normal. The pattern of LV dysfunction was apical akinesis in one, and apical septal hypokinesis in the other. Both patients recovered within 4 weeks with normalization of LVEF. The concurrence of doxorubicin in the chemotherapeutic regimen, asymptomatic presentation, and higher LVEF were unique features noted in the combretastatin-related TCM cases.

### Rituximab

Rituximab is a commonly used monoclonal anti-CD20 antibody with a wide spectrum of applications, ranging from use in autoimmune disorders to treatment of hematologic disorders. Its use in treating follicular non-Hodgkin’s lymphoma, chronic lymphocytic leukemia (CLL), and autoimmune diseases like rheumatoid arthritis is well known, along with its role as an anti- rejection agent after organ transplant.

One case of rituximab-associated TCM has been reported in the literature. Ng et al. [69] published a case of a 66-year-old man with CLL who developed dyspnea and flushing after rituximab infusion. The patient was asymptomatic; however, ECG demonstrated ST elevation, and troponin was elevated. Angiography revealed no significant CAD, and echocardiogram showed LVEF of 40% with wall motion abnormalities suggestive of TCM. LVEF recovery could not be documented as the patient died of non-cardiac cause.

Rituximab has been reported to cause adverse cardiac events including arrhythmia and rarely myocardial infarction. However, its detailed cardiotoxicity profile and effects on cardiac function are not well described [32]. Two postulated mechanisms explaining the pathophysiology of rituximab-induced TCM have been proposed: 1) Neurohormonal activation that leads to stimulation of the sympathetic system leading to coronary vasospasm, and 2) direct cardiotoxic effect leading to ventricular dysfunction [32]. Diffuse reticulin fibers along with increased levels of transforming growth factors (TGF)–β were present in cardiac myocytes after treatment with rituximab. Cardiac events have been reported during later infusions of rituximab, possibly as a result of reticulin fiber accumulation in cardiac myocytes over time [70].

### Tyrosine kinase inhibitors

Tyrosine kinases are enzymes associated with the cytoplasmic domains of growth factor receptors and play a pivotal role in cellular regulation. If mutated or hyper-expressed, these kinases can lead to downstream hyperactivation, leading to overgrowth of cells. Thus, tyrosine kinases are an excellent target for cancer drugs [71].

Axitinib [72] is an oral selective second-generation tyrosine kinase inhibitor (TKI) which blocks angiogenesis and tumor growth by inhibiting vascular endothelial growth factor receptors (VEGFR-1, VEGFR-2, and VEGFR-3). Sunitinib [73] exhibits antitumor and anti-angiogenic properties by inhibiting multiple receptor tyrosine kinases, including platelet-derived growth factors (PDGFRα and PDGFRβ), vascular endothelial growth factors (VEGFR1, VEGFR2, and VEGFR3), FMS-like tyrosine kinase-3 (FLT3), colony-stimulating factor type 1 (CSF-1R), and glial cell-line-derived neurotrophic factor receptor (RET). Both these drugs have been approved for use in the treatment of advanced renal cell carcinoma. Cardiovascular adverse effects of these TKIs include hypertension, hypertensive crisis, and thromboembolic events.

Recently, TKIs have been associated with the development of TCM. Numico et al. [74] reported a case of a 57-year-old woman undergoing treatment of clear cell renal carcinoma with sunitinib who presented with chest pain and ECG changes and was found to have a reduction in LVEF to 15–20% in the absence of significant coronary artery disease. Her LV function recovered to normal 3 months later. Similarly, axitinib has been reported to cause TCM in a case report of a 71-year-old woman with chest pain and anterior ST segment elevation on ECG, who developed LVEF reduction to 20–25% in the absence of obstructive CAD on angiogram. Her LVEF improved 3 weeks later [33].

Ibrutinib, which is an inhibitor of Bruton’s tyrosine kinase used to treat B cell cancers, such as mantle cell lymphoma, chronic lymphocytic leukemia, and Waldenstrom’s macroglobulinemia, has been reported to cause Takotsubo cardiomyopathy. Gize et al. described a case of a 53-year-old woman with non-small-cell lung cancer, who developed TCM after treatment with ibrutinib. Echocardiogram demonstrated marked left ventricular dysfunction with mid-ventricular hypokinesis and apical hyperkinesis [75].

### Immune checkpoint inhibitors

Immune checkpoint inhibitors are the poster child of translational tumor immunology and immunotherapy. These drugs consist of human antibodies directed against cytotoxic T lymphocyte antigen-4 (CTLA-4) and programmed death-1 (PD-1) [76]. Immune checkpoint inhibitors, which include ipilimumab, pembrolizumab and nivolumab to name a few, lead to the abrogation of checkpoints on the immune system, causing increased tumor destruction by the host immune cells. The advent of these therapies has changed the face of treatment for melanoma [77] as well as some solid tumors [34].

Despite important clinical benefits, immune checkpoint inhibition is associated with a unique spectrum of side effects termed immune-related adverse events (irAEs) [35]. These side effects can range from dermatologic, gastrointestinal, hepatic, endocrine, and to other less common inflammatory events. Immune-mediated myocarditis [36] has been reported, in some cases, requiring treatment with advanced heart failure therapies. To date, there has been one report of a TCM-like syndrome characterized by apical ballooning and hyperdynamic basal segment in the setting of nonobstructive CAD on angiogram in a metastatic melanoma patient treated with ipilimumab [78]. However, in this case, myocarditis could not be definitively excluded.

### Miscellaneous drugs

Khanji et al. [37] reported a case of trastuzumab-induced TCM in a 50-year-old postmenopausal woman with invasive ductal carcinoma of the breast undergoing treatment with trastuzumab, docetaxel, and carboplatin. During the eleventh cycle of trastuzumab, she presented with chest pain, ECG changes, and elevated troponin. She was found to have a reduced LVEF with dyskinesis of the mid LV myocardium with normal apical and basal wall motion on ventriculography, consistent with an atypical variant TCM or “reverse Takotsubo”. She had a normal coronary angiogram. Her LV function normalized in 4 weeks. Trastuzumab is associated with ventricular dysfunction in 3.2 to 18.6% of cases, which is largely reversible and is described as “Type II” chemotherapy-related cardiotoxicity [56]. This cardiotoxicity is characterized by ventricular dysfunction secondary to interference with normal growth, repair, and survival of cardiomyocytes. It is currently unclear whether and how trastuzumab-induced ventricular dysfunction differs from TCM in clinical characteristics and pathogenesis. The safety of trastuzumab re-challenge once the LVEF normalizes is also an area that needs further exploration.

Goel et al. [79] described the case of a 55-year old-man diagnosed with non-M3 Acute Myeloid Leukemia (AML) who developed TCM with daunorubicin and cytarabine chemotherapy 6 days into chemotherapy. Another case of daunorubicin-induced TCM was reported in a patient with multiple myeloma [80]. Anthracyclines remain among the most widely used anti-cancer drugs for solid and hematologic malignancies. The mechanism of anthracycline cardiotoxicity is postulated to involve cardiac damage induced by DNA double-strand breaks, reactive oxygen species, and mitochondrial dysfunction [81]. Cardiac myocytes are susceptible to such damage given the high mitochondrial density and dearth of anti-oxidant enzymes leading to the irreversible loss of cardiac myocytes, known as “Type I” chemotherapy-related cardiotoxicity [82]. Whether anthracyclines also cause a transient, reversible cardiomyopathy akin to TCM remains to be determined.

## Discussion

In our review of the literature, we found 25 cases of chemotherapy-associated TCM. Among the patients in the reviewed studies, the mean age was 59 years (range 14 to 83 years). Although TCM predominantly affects postmenopausal women in the general population, our review revealed a similar incidence of TCM induced by cancer drug in men and women. Among the analyzed cases, none had a previous history of CAD. Most patients (54%) presented with chest pain. 75% of patients had ST segment deviation, and the rest had at least T wave changes on presentation. The mean LVEF on presentation was 27% (normal range 55–75%). In the studies we examined, myocarditis was excluded in only 5 patients: 4 patients by cardiac MRI (CMR) and 2 patients by endomyocardial biopsy (1 patient had both CMR and biopsy).

In the review of case reports, most patients had an improvement in LVEF to normal within a mean duration of 1 month (range 5 days to 3 months), which demonstrates reversibility of this condition and response to adequate therapy. Good prognosis is also supported by the finding that all patients survived except three, among whom one patient had a cardiac death, while the other two deaths were attributed to a non-cardiac cause (cancer progression in one case and pathological fracture in the other). Most patients did not require advanced therapies, such as intra-aortic balloon pump (IABP) or mechanical circulatory support, except for two patients, in whom IABP was used.

Consistent with the literature, we found a wide range of cancer drugs to be associated with TCM – most commonly 5-FU (42%) followed by capecitabine (21%). Other anti-neoplastic agents reported to be associated with TCM were bevacizumab, combretastatin, rituximab, tyrosine kinase inhibitor (axitinib, sunitinib, and ibrutinib), trastuzumab, daunorubicin, and ipilimumab. Most often, chemotherapy-associated TCM occurred during the first cycle of chemotherapy (17 out of 25 cases; 68%). Overall, dose was poorly mentioned in the studies. Thus, it is difficult to decipher any correlation between the dose of chemotherapy and TCM.

A retrospective analysis conducted at M.D. Anderson Cancer Center showed that the most common cancers associated with TCM were lymphoproliferative neoplasms (23.3%), followed by gastrointestinal (17%), ovarian (13.3%) and breast cancer (10%) [75]. Our review revealed a preponderance of gastrointestinal, head and neck, and breast cancers in the case reports of drug-induced TCM.

Patients with cancer are at an elevated risk of developing TCM because of emotional and psychosocial stress, physical stress of surgery and radiation treatment, and biochemical stress of systemic anti-neoplastic therapy. The true incidence of TCM in patients with malignancy receiving chemotherapy remains unknown. An analysis of chemotherapy-related adult hospitalizations using the National Inpatient Sample database (2010 to 2014) revealed an overall increasing nationwide trend in TCM incidence in patients receiving chemotherapy [83].

Patients with malignancy not only have an increased risk for TCM development, they experience worse outcomes compared to patients with TCM in the absence of malignancy. Based on National Inpatient Sample (NIS) analysis study [84], TCM with coexisting malignancy had a significantly higher mortality (13.8 vs. 2.9%, *p* < 0.0001), length of stay (7 vs. 4 days, p < 0.0001) and total charges ($29,291 vs. $36,231, p < 0.0001), compared to those with no malignancy. Thus, early recognition and prompt initiation of appropriate treatment may help lower mortality and reduce health care costs in these patients.

The management of hemodynamically stable patients with TCM involves the initiation of standard guideline-directed medical therapy (GDMT) for heart failure with reduced ejection fraction, which includes beta blocker therapy and an angiotensin converting enzyme (ACE) inhibitor (or angiotensin II receptor blocker [ARB]) and diuretics as necessary to treat volume overload [85]. In patients with left ventricular outflow tract (LVOT) obstruction, care should be taken to avoid volume depletion and vasodilator therapy. In addition, hypotension associated with significant LVOT obstruction should not be treated with inotropic agents, such as dopamine and dobutamine, because they can worsen the degree of obstruction [38].

There are no prospective clinical trial data to guide the optimal duration of medical therapy for patients with TCM. In general, GDMT is continued until there is recovery of systolic function, which typically occurs in 1 to 4 weeks [86]. Whether GDMT can be discontinued after normalization of LVEF remains unknown. However, results from a recent randomised trial, TRED-HF, which examined the effect of GDMT withdrawal in patients with previous dilated cardiomyopathy who have recovered their LVEF support the value of indefinitely continuing medical therapy [87]. In this study, 44% of patients assigned to treatment withdrawal developed a relapse of cardiomyopathy during the first 6 months. Given the high rate of relapse, it is recommended to continue treatment indefinitely for dilated cardiomyopathy. For patients with TCM, as well, indefinite continuation of medical therapy is preferable since the annual rate of recurrence is estimated at 1.5% [88]. Observational data suggest that the use of an ACE inhibitor or ARB was associated with improved survival at 1 year [88]. More prospective clinical trials are needed to define the optimal therapy for TCM.

In patients who require further cancer treatment, re-challenging with the culprit anti-neoplastic therapy poses a major clinical dilemma. There is limited data on the safety of reintroducing the chemotherapeutic agent. In a retrospective analysis of 30 patients with cancer and stress-induced cardiomyopathy treated at the MD Anderson Cancer Center, 21 patients required ongoing cancer treatment. Among them, 16 were able to safely resume chemotherapy after normalization of LVEF without recurrence of TCM. Median time to resume cancer treatment was 20 days after TCM [76]. Among the 25 case reports we reviewed, re-challenge was pursued in two cases, both of which involved 5-FU. In one case, re-challenge with half the dose was tolerated. However, when the dose was escalated to the full dose, the patient suffered a cardiac arrest [18]. In another case, re-challenge with the full dose was tolerated for 4 cycles before cardiac arrest developed [44]. Further studies are required before we can make any conclusions on re-challenge.

With this review of the literature reporting chemotherapy-associated TCM, we are left with two important unanswered questions [1]: Do these reported cases truly represent Takotsubo cardiomyopathy, or rather chemotherapy-induced myocarditis (since myocarditis was excluded in only 5 patients) or a different type of chemotherapy-related reversible non-ischemic cardiomyopathy? [2] If these cases truly represent TCM, can we conclusively attribute the cancer drug as the etiologic agent for TCM?

In a patient presenting with chest pain and dyspnea during or after their cancer treatment, the differential diagnosis includes acute coronary syndrome, venous thromboembolism, acute heart failure due to cardiotoxicity, myocarditis, and Takotsubo syndrome. Diagnostic procedures include echocardiogram, myocardial necrosis markers, natriuretic peptides, CT angiogram of the chest as necessary to rule out pulmonary embolism, and coronary angiogram to exclude acute coronary plaque rupture. In addition, CMR or endomyocardial biopsy is considered to exclude myocarditis. The classic wall motion abnormalities of the LV apical and mid segments which extend beyond a single coronary distribution or the typical appearance of “apical ballooning” provide a clue to the diagnosis of TCM. In the cases of TCM where the LV contractile dysfunction is diffuse or global, it is difficult to distinguish Takotsubo syndrome from another type of chemotherapy-related cardiac dysfunction. There is no universal definition of cardiotoxicity; however, most organizations have defined cardiotoxicity as an asymptomatic decline in LVEF ≥10% to a final ejection fraction that is less than the lower limit of normal; i.e. LVEF 50 to 55%. Cardiotoxicity also includes a less significant decline in LVEF in the presence of signs and symptoms of heart failure [89, 90].

In a patient with cancer, a variety of other factors may contribute to the development of TCM: the emotional stress of a cancer diagnosis; the physical stress of surgery, radiation treatment, and chemotherapy; and the inflammatory state of the cancer itself. The exact trigger for TCM is often difficult to identify. At this time, one cannot conclude more than a mere association of these cancer drugs with TCM. Additional studies are required to investigate the role of chemotherapeutic and novel targeted agents in inducing this particular type of cardiomyopathy.

## Conclusion

Patients receiving multi-modality treatment for their cancer with surgery, radiation and chemotherapy are at an increased risk for developing TCM. When this condition occurs in a patient with malignancy, it is associated with worse outcomes compared to the general population [84]. Therefore, it is essential for medical professionals to recognize, monitor, and treat this condition appropriately. Although the prognosis from TCM is generally good, this condition can lead to an interruption in chemotherapy, which may adversely affect oncologic outcome. Whether the drug-induced cardiomyopathy truly represents TCM or another type of chemotherapy-related reversible cardiotoxicity is an important topic for further investigation. In addition, if the cardiomyopathy is indeed part of the Takotsubo syndrome, whether the cancer drug is the offender, or just a bystander, is a critical question that needs to be considered in a case-by-case scenario.

## Data Availability

Data sharing is not applicable to this article as no datasets were generated or analysed during the current study.

## Abbreviations

5-FU: 5-Fluorouracil
ACE: Angiotensin converting enzyme
ACS: Acute Coronoary Syndrome
AML: Acute Myeloid Leukemia
ARB: Angiotensin II receptor blocker
CAD: Coronary artery disease
CLL: Chronic lymphocytic leukemia
CMR: Cardiac MRI
ECG: Electrocardiogram
FOLFIRI: 5-FU, folinic acid and Irinotecan
FOLFOX: 5-FU, folinic acid and oxaliplatin
GDMT: Guideline-directed medical therapy
IABP: Intra-aortic balloon pump
LV: Left ventricle
LVEF: Left ventricular ejection fraction
LVOT: Left ventricular outflow tract
MI: Myocardial infarction
TCM: Takotsubo cardiomyopathy
TKI: Tyrosine kinase inhibitor
VEGF: Vascular endothelial growth factor
VEGFR: Vascular endothelial growth factor receptor
VT: Ventricular tachycardia
